# Pharmacokinetics and Safety of a Novel Oral Liquid Formulation of 13-*cis* Retinoic Acid in Children with Neuroblastoma: A Randomized Crossover Clinical Trial

**DOI:** 10.3390/cancers13081868

**Published:** 2021-04-14

**Authors:** Gareth J. Veal, Deborah A. Tweddle, Johannes Visser, Julie Errington, Helen Buck, Josephine Marange, Jon Moss, Shiju Joseph, Hussain Mulla

**Affiliations:** 1 Newcastle University Centre for Cancer, Translational & Clinical Research Institute Newcastle University, Newcastle upon Tyne NE2 4HH, UK; gareth.veal@newcastle.ac.uk (G.J.V.); deborah.tweddle@newcastle.ac.uk (D.A.T.); julie.errington@newcastle.ac.uk (J.E.); 2 Great North Children’s Hospital, Newcastle upon Tyne NE1 4LP, UK; 3 Cambridge University Hospitals NHS Foundation Trust, Cambridge CB2 0QQ, UK; johannes.visser@nhs.net; 4 ClinPsy Limited, St. Albans, Hertfordshire AL3 5HN, UK; drhelen.buck@yahoo.co.uk; 5 Nova Laboratories Limited, Leicester LE18 4YL, UK; josephine.marange@novalabs.co.uk; 6 BAST Inc Limited, Loughborough Innovation Centre, Loughborough LE11 3AQ, UK; jjmoss@bastinc.eu (J.M.); sjoseph@bastinc.eu (S.J.); 7 Department of Pharmacy, University Hospitals of Leicester NHS Trust, Leicester LE3 9QP, UK

**Keywords:** neuroblastoma, 13-CRA, pharmacokinetics, liquid

## Abstract

**Simple Summary:**

The availability of a child-friendly drug formulation can be an important determinant of therapeutic success. 13-*cis*-retinoic acid (13-CRA) is a key component of high-risk neuroblastoma treatment protocols, and yet there is no such formulation available in any country worldwide. Typically, parents have an onerous and potentially hazardous daily task of extracting 13-CRA from capsules. We have developed a novel oral liquid formulation, which is stable and permits accurate daily dosing. This study compared the pharmacokinetics (PK), safety and palatability of the liquid formulation to the current capsule extraction approach to dosing. The liquid formulation demonstrated superior bioavailability, increasing the likelihood of achieving therapeutic levels, which in previous studies had proven difficult with 13-CRA extracted from capsules. The adverse effect profile was as expected for 13-CRA and was similar between the two formulations. Feedback on palatability was good with both dosing approaches, but parents found daily dosing much easier with the liquid formulation.

**Abstract:**

(1) Background: 13-*cis*-retinoic acid (13-CRA) is a key component of neuroblastoma treatment protocols. This randomized crossover study compares the pharmacokinetics (PK), safety and palatability of a novel oral liquid formulation to the current method of extracting 13-CRA from capsules. (2) Methods: Pharmacokinetics was evaluated in two consecutive treatment cycles. Patients were randomized to receive either liquid or capsule formulation on cycle 1 and then crossed over to the alternative formulation on cycle 2. The daily dose was 200 mg/m^2^, reduced to 160 mg/m^2^ in patients with weight ≤ 12 kg. (3) Results: A total of 20 children, median (range) age 4.3 (1–11.6) y were recruited. Pharmacokinetic data were pooled and a population model describing the disposition of 13-CRA and 4-oxo-13-CRA was developed. Bioavailability of the liquid formulation was estimated to be 65% higher (95% CI; 51–79%) than the extracted capsule. CmaxSS and AUC(0-12)SS estimates were also significantly higher; mean (95% CI) differences were 489 (144–835) ng/mL and 3933 (2020–5846) ng/mL·h, respectively (*p* < 0.01). There were no significant differences in reported adverse effects. Parents found dosing considerably easier with liquid formulation. (4) Conclusions: The pharmacokinetics, safety and palatability of a new liquid formulation of 13-CRA compares favorably to 13-CRA extracted from capsules. Clinical Trial Registration: clinicaltrial.gov NCT03291080.

## 1. Introduction

Neuroblastoma (NBL) is an aggressive childhood cancer with an annual EU and US incidence of 850 and 600 cases per year, respectively. Approximately 45–50% of these children will be diagnosed with high-risk neuroblastoma (HRNBL), defined by metastatic disease in those over the age of 12 to 18 months and *MYCN* amplification at any age [1]. 

First-line therapy in children who present with HRNBL consists of multimodal therapeutic regimens including multi-agent induction chemotherapy, surgical resection of the primary tumor, high-dose chemotherapy with autologous hematopoietic stem cell rescue, radiotherapy to the site of the primary tumor and post-consolidation treatment with oral 13 *cis*-retinoic acid (13-CRA, also known as isotretinoin) and/or immunotherapy [1]. However, as about 40% of HRNBL patients relapse, there is not only an urgent need for more efficacious treatment strategies, but also a need to optimize existing therapeutic approaches. 

13-CRA is one of several stereoisomers of retinoic acid, the main biologically active derivative of vitamin A, and has been used as a cancer chemopreventive agent due to its ability to induce cell differentiation, inhibit proliferation and induce apoptosis [2]. An initial randomized clinical study utilizing low continuous dosing with 100 mg/m^2^/day 13-CRA showed no clinical benefit [3]. However, in a subsequent randomized trial of HRNBL patients, myeloablative chemotherapy and autologous hematopoietic stem cell transplant resulted in a significantly better 5-year event-free survival (EFS) and overall survival (OS) than non-myeloablative chemotherapy; in both groups high-dose 160mg/m^2^/day pulsed dosing 13-CRA, independently improved OS [4]. This suggests that optimizing the 13-CRA dose is an important determinant of efficacy. 

13-CRA is now considered as standard maintenance treatment alongside immunotherapy and is administered by the enteral route, at a dose of 80 mg/m^2^ twice daily (160 mg/m^2^/day), in 2-week cycles for 6 months. However, it is still the case that the only marketed formulation of 13-CRA is an oral capsule with an indication for severe acne. Young children with NBL are often unwilling and/or unable to swallow capsules. Consequently, carers are asked to open and extract the 13-CRA from the capsule and mix with foods prior to administration. This method of administration was shown to result in up to 20-fold variability in patient drug exposure, with some patients experiencing substantially lower plasma concentrations, potentially increasing the risk of treatment failure [5,6]. Protocol doses were subsequently increased to 200 mg/m^2^/day for children unable to swallow intact capsules. Furthermore, 13-CRA is a potential teratogen and it must be used and handled with care. The practice of extracting drug from capsules puts mothers of childbearing age at risk of accidental exposure to 13-CRA. While this situation is clearly unacceptable, both from a treatment point of view and a safety perspective, it is a situation currently faced by families of children with HRNBL around the world.

To overcome the challenge of dosing accuracy and palatability and to minimize the teratogenic risk for women of childbearing potential with the current method of manipulation of capsule, a convenient, ready-to-use, multi-dose, oral liquid formulation of 13-CRA has been developed. The aims of this open label, randomized, multiple-dose, crossover study were to compare the pharmacokinetics (PK), safety and palatability of the newly developed oral liquid formulation with the method of extracting 13-CRA from capsules.

## 2. Materials and Methods

### 2.1. Study Patients

The trial (ClinicalTrials.gov NCT03291080) was conducted in accordance with the principles of the International Conference on Harmonization–Good Clinical Practice guidelines and was approved by the UK Medicines and Healthcare Products Regulatory Agency (MHRA) and the North East–Newcastle and Tyneside 1 Research Ethics Committee. 

Children were recruited from 12 pediatric oncology centers across the UK (see list of participating sites in acknowledgements). Eligibility for inclusion in the trial included: diagnosis of HRNBL or unresectable unfavorable histology intermediate-risk neuroblastoma, the latter age ≥ 18 months at diagnosis; patients who were scheduled to receive at least two treatment cycles of 13-CRA; patients who could not swallow intact 13-CRA capsules (i.e., required extraction of 13-CRA from the capsules). Exclusion criteria were appropriate for the study design and accounted for known potential safety concerns and drug interactions that could compromise the PK analysis.

No formal sample size calculations were performed. The target sample size was considered appropriate based on previous experience of population PK modelling studies. In order to develop a robust population PK model (i.e., precisely estimated parameters), PK concentration data from a minimum of >150 blood samples obtained from 20 (minimum 12) patients were required. 

**Figure 1:**
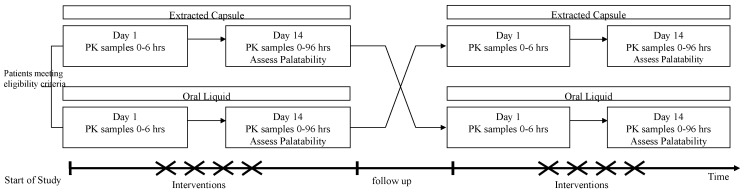
Schematic of study design.

The relative bioavailability and PK of 13-CRA oral liquid (test product) and capsule-extracted 13-CRA (reference product) were evaluated over a consecutive 2-month period of a 6-cycle (6-month) treatment phase. Patients were randomized to receive either oral liquid or extracted capsule formulation in cycle 1 and then crossed over to the alternative formulation in cycle 2. Randomization was carried out once eligibility had been confirmed and the next scheduled treatment visit arranged. This was done centrally by Nova according to a randomization list consisting of allocated treatment sequences, generated by the trial statistician. Each treatment cycle lasted 14 days, followed by a 14-day break between treatment cycles, as per standard treatment protocols (Figure 1) and negating any carryover effects.

13–CRA was prescribed according to local treatment protocols at each site and independent of the study. The dose administered was 200 mg/m^2^/day (in two divided doses) for both test and reference product. Patients with a body weight of ≤12 kg received a dose of 160 mg/m^2^/day.

### 2.2. Adverse Event Reporting

All adverse events (AE) were recorded and graded as per Common Terminology Criteria for Adverse Events (CTCAE, version 4.03) during the trial and for 14 days follow-up. Oropharyngeal tolerability and symptoms (dry skin, peeling skin, cracked lips and dry eyes) were separately assessed at baseline (prior to each treatment cycle) and on Day 14 using a four-point scale: 0 = none, 1 = mild, 2 = moderate and 3 = severe. Patients and parents were provided with a diary to record any oropharyngeal events during treatment. An independent Data Safety Monitoring Committee (DSMC) reviewed the interim safety and PK data at periodic intervals. 

### 2.3. Palatability and Acceptability

At the end of each of the two treatment cycles the palatability and acceptability of the two methods of administering 13-CRA were assessed using a previously reported questionnaire tool [7]. This tool uses a combination of a 0–100 mm visual (hedonic) analogue scale (0 mm being “bad” and 100 mm being “good”) and verbal responses to perception of taste, smell and ease of drug administration. This assessment was conducted face-to-face. In the case of patients <6 years of age, the view of one or both parents was surveyed. For patients >6 years of age, the views of the patient and the parents were surveyed.

Analysis of safety and palatability data was of a descriptive nature, using appropriate summary statistics (e.g., mean, SD, median, minimum, maximum) or frequency distributions (n%) by age group.

### 2.4. PK Blood Sampling and Analysis

Blood samples for PK analysis were taken on Day 1 and Day 14 of each of the two treatment cycles. Patients were administered morning doses in the clinic and post-dose samples were taken at 0.5, 1, 1.5, 2, 3, 4, 5 and/or 6 h. The 2 h and 6 h samples were mandatory, with at least three further samples (where possible) selected over 0.5 to 5 h. In addition, an additional sample at 24–48 h following their final dose on Day 14 of each of the two treatment cycles was obtained in patients for whom this was practicable. Blood samples (2 mL) were collected in heparinized tubes and centrifuged at 1200 g for 5 min at 4 °C. Plasma was separated and frozen at −20 °C prior to analysis. All blood and plasma samples were wrapped in aluminum foil to protect them from light, and all sample handling was carried out in dim light. 

Quantification of 13-CRA and 4-oxo-13-CRA (active metabolite) levels was carried out by LC/MS/MS analysis using an API4000 triple quadrupole mass spectrometer from SCIEX (Foster City, CA, USA), attached to a Prominence series HPLC system (Shimadzu, Milton Keynes, Buckinghamshire, UK). A Supelcosil ABZ+Plus (10 cm × 2.1 mm, 3 µm) column (Sigma Aldrich, Dorset, UK) with a Phenomenex Security guard containing a C18 cartridge (4 × 2 mm) was utilized for sample separation following acetonitrile extraction. Mobile phase A (MP A) consisted of 0.1% formic acid in Milli-Q water and MP B was 0.1% formic acid in acetonitrile. The HPLC system was set at a constant flow rate of 0.4 mL/min and run at ambient temperature under gradient conditions: step 1: 40% MP A to 5% over 6 min; step 2: 5% MP A to 40% over 0.5 min; step 3: constant for 3.5 min. 

The assay was validated with regards to specificity, linearity, reproducibility, carry over, recovery and stability of the analytes according to EMA guidelines. The assay had a limit of detection (LOD) of 5 ng/mL and a lower limit of quantification (LLOQ) of 20 ng/mL for both 13-CRA and 4-oxo-13-CRA, and exhibited within- and between-run coefficients of variation and bias below 15%. Quality Control (QC) samples for both the parent drug and metabolite were included in each assay. Calibration curves were linear between 20–1000 ng/mL with r^2^ values > 0.99. Samples containing concentrations of analytes above the linear range were diluted with blank plasma.

### 2.5. PK Modelling Analysis

PK disposition and systemic exposure parameters (clearance, volume of distribution, relative bioavailability, Cmax, AUC) were estimated using a population PK modelling approach. Development of a population PK (popPK) model for 13-CRA and 4-oxo-13-CRA was performed with the nonlinear mixed effects modelling program NONMEM (ICON Development Solutions, version 7.4 [8]). The FOCE model parameter estimation method was used at all times, except when concentrations below the LLOQ were included in the dataset, at which point the M3 method along with Monte Carlo importance sampling (IMP) estimation method was used. Data file preparation, model evaluation and all other graphical and statistical analyses were executed using the R software, version [9]. Simulations for visual predictive checks (VPC) were executed using NONMEM [10]. Simulations of exposure variables on the basis of the final selected popPK model were done in R using the “RxODE” library [11]. The full modelling methodology can be found in Supplement File S1.

## 3. Results

### 3.1. Patients

The study was initiated in June 2018 and recruitment stopped in July 2019 once the target sample size was reached. Parents of 22 children diagnosed with neuroblastoma consented to take part in the study and 20 were screened and entered the study; 18 patients had a diagnosis of high-risk neuroblastoma, 2 with intermediate-risk neuroblastoma. 

Eleven subjects received liquid formulation in the first cycle followed by capsule-extracted 13-CRA in the next cycle and vice versa for the remaining 9 subjects. Twelve patients received the medicine via a nasogastric or gastrostomy tube, 8 patients received medication orally. Compliance with therapy was excellent in all patients as recorded on diary cards. Only one patient withdrew early due to a serious adverse event. 

Table 1 summarizes the demographics of the study population: 25% of the subjects were female and baseline median (range) age and body weight were 4.3 (1–11.6) y and 14.2 (9.3–38.9) kg, respectively. The median measured BSA at baseline was 0.6 m^2^ (range 0.4 to 1.3) and the median BMI at baseline was 15.8 kg/m^2^ (range 13.4 to 18.2). Eighteen patients received a dose of 200 mg/m^2^, 2 patients received 160 mg/m^2^.

### 3.2. Pharmacokinetic Model and Relative Bioavailability

**Figure 2:**
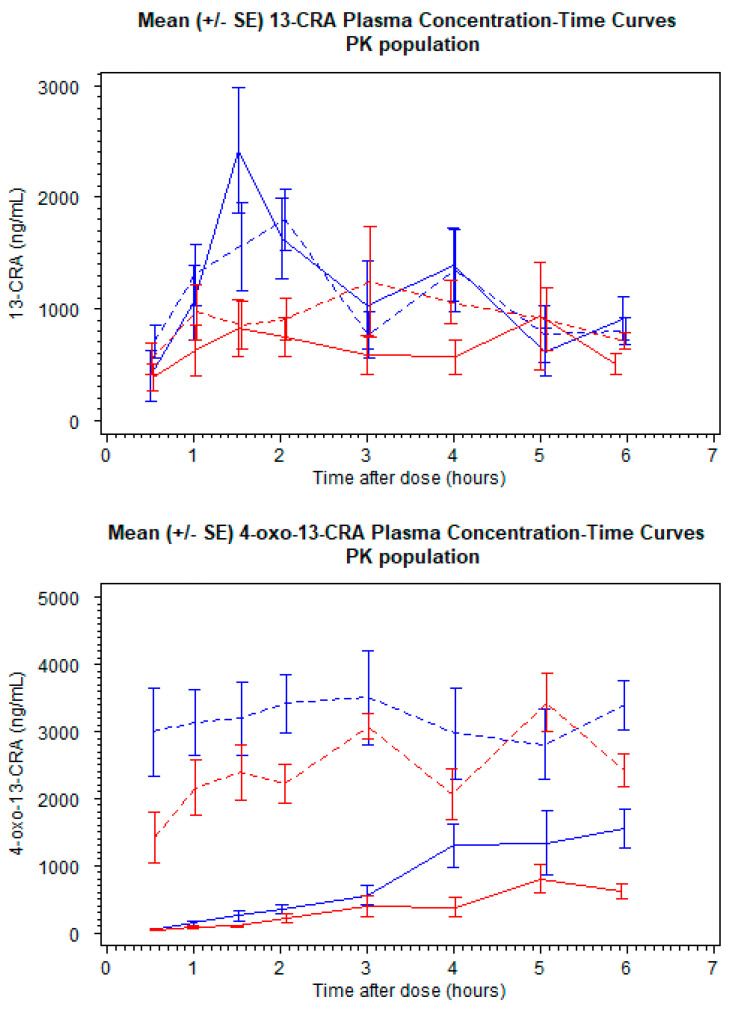
Mean (SE) plasma concentration versus time profiles for 13-*cis*-retinoic acid (13-CRA) (upper panel) and 4-oxo-13-CRA (lower panel). Blue lines are oral liquid, red lines are extracted capsule. Continuous lines are Day 1, interrupted lines are Day 14.

A total of 20 patients provided 373 plasma samples that were above the LLOQ for 13-CRA, and 262 concentrations above the LLOQ for 4-oxo-13-CRA from 15 patients. Twenty-one 13-CRA concentrations and twenty-eight 4-oxo-13-CRA concentrations were below the LLOQ. The mean (SD) plasma concentration profiles for the two formulations are shown in Figure 2. A popPK parent–metabolite model to describe 13-CRA and 4-oxo-13-CRA was developed from pooled plasma concentration data. LLOQ concentrations were included in the final model development phase.

It was observed for the liquid formulation that there was a time delay in the absorption phase. To compensate for over-prediction by the model during the early time-points, transit compartments were included, although no such time delay was necessary for the capsule-extracted formulation. Furthermore, whilst a one-compartment disposition model with a first-order absorption rate constant for liquid and capsule-extracted formulation, along with two transit compartments and relative bioavailability estimated for liquid formulation adequately described the drug behavior during the first few hours after dosing, an under-prediction was observed for later time points. The probable cause for this under-prediction was presumed to be the biphasic behavior of 13-CRA, as it is known to have enterohepatic recirculation properties [12]. To incorporate this behavior, a second compartment was included and intercompartmental rate constants were estimated. This second compartment imitates the gall bladder, wherein 13-CRA is returned to the circulatory system of the body at patient meal times. In the absence of meal-time information, it was assumed that the enterohepatic recirculation could be adequately described by a 2-compartment model (Figure S1). The inclusion of this additional compartment dropped the objective function value (OFV) significantly and the under-prediction of the later time-points was reduced. Individual parameter values from the model for 13-CRA were carried forward in order to separately model the 4-oxo-13-CRA concentrations. The obtained individual models were then combined and both 13-CRA and 4-oxo-13-CRA data were simultaneously modelled (see Supplement File S1).

Only allometric scaling by weight (to a fixed exponent of 0.75) was found to be an influential covariate on the kinetics of 13-CRA and 4-oxo-13-CRA. The parameter estimates from the final model (Table S1), as well as the diagnostic plots (Figures S2–S4) are presented in the Supplement (File S1). 

### 3.3. Model Estimates of Systemic Exposure

The bioavailability of the oral liquid formulation of 13-CRA relative to the capsule-extracted 13-CRA was estimated by the model to be 65% higher (95% CI 51–79%).

**Figure 3:**
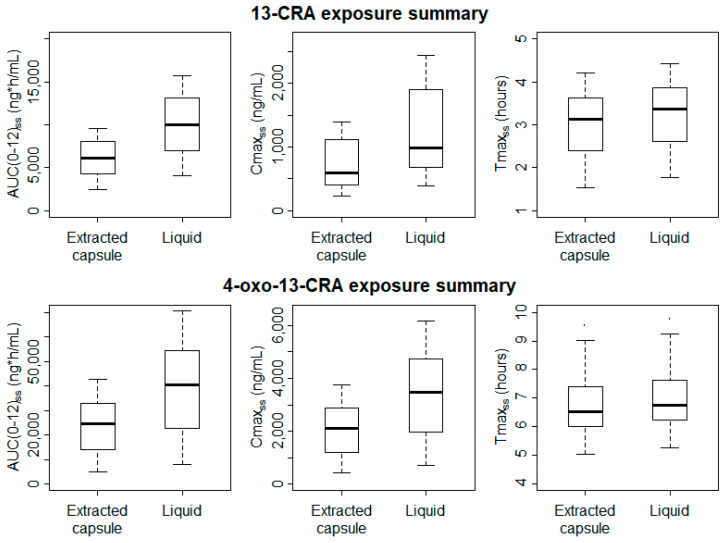
The plots above compare the individual model-predicted AUC_(0-12)ss_, Cmax_ss_ and Tmax_ss_ (left-to-right) of 13-CRA (upper panel) and 4-oxo-13-CRA (lower panel). Individual values are summarized as vertical box-and-whisker plots, with the box covering the interquartile range (IQR), the median shown by the thick black line and with whiskers extending to 1.5 times the IQR.

Significantly higher Cmax_SS_ and AUC_(0-12)SS_ values were estimated for both 13-CRA and 4-oxo-13-CRA when patients were administered the new oral liquid formulation (Figure 3); for 13-CRA, mean (95% CI) difference between the two formulations was 489 (144–835) ng/mL and 3933 (2020–5846) ng/mL·h, respectively (*p* < 0.01). Despite the slight delay in the onset absorption of the oral liquid, there was no difference in the Tmax_ss_ (*p* > 0.9).

There is no indication that the variability in exposure of either 13-CRA or 4-oxo-13-CRA is reduced when administration is via the oral liquid formulation compared to the capsule-extracted 13-CRA. However, it is difficult to make robust conclusions regarding inter-subject variability with a small population of 20 pediatric patients.

### 3.4. Safety and Tolerability

There was a total of 35 drug-related events (includes definite, probable or possible classification) reported by 13 patients in the oral liquid cycle compared to 26 related events reported by 9 patients in the extracted capsules cycle (Table 2). The majority of AEs were mild in severity. There were no drug-related Grade 3+ treatment-emergent AEs or deaths. There was no correlation between adverse events and Cmax/AUC.

“Chapped lips” was reported by 8 (40%) patients in the oral liquid cycle and 5 (25%) patients in the extracted capsules cycle. Skin and subcutaneous tissue disorders such as dry skin were recorded by 9 (45%) patients in the oral liquid cycle and 11 (55%) patients in the extracted capsules cycle. “Dry eye” was also frequently reported; 4 (20%) patients in the oral liquid cycle and 2 (10%) patients in the extracted capsules cycle.

Two serious adverse events (SAE), gastrointestinal bleed with hematemesis leading to cessation of therapy, and acute renal failure, were considered possibly related in two (10%) patients during the oral liquid cycle compared to none in the extracted capsule cycle. However, there was no suggestion this was influenced by the formulation. 

Overall, there were no significant differences between the two treatment cycles in terms of known AE of 13-CRA, expected events for HRNBL patients and overall safety during the study. The independent DSMC reviewed the interim safety and PK data and recommended that the study continue without modification at all review meetings. 

### 3.5. Palatability and Acceptability

The results are presented in Table 3. The majority of children found the taste and smell of both the liquid and the capsule-extracted 13-CRA to be “fairly good” or “good”, with only one subject reporting a residual aftertaste with both formulations. 

As anticipated, there was a large difference in terms of the perception of ease of preparation and administration. The liquid formulation was perceived as being easier to take (85.7% reporting it is “easy to take all of the time” and 14.3% reporting it to be “easy to take most of the time”). In contrast, with the extracted capsules, only 50.0% reported it is “easy to take all of the time”, and 18.8% reported it as being “not at all easy to take”. The vast majority of parents found the liquid easier to administer, with no parent reporting the liquid difficult to administer. In contrast, 73.3% of parents found it “not easy” to “extremely difficult” to extract 13-CRA from capsules.

## 4. Discussion

The importance of child-appropriate formulations of medicines has been highlighted continuously by healthcare professionals, regulatory authorities and international organizations including the WHO [13]. 13-CRA has been a key component of the HRNBL treatment protocol for almost 20 years and yet the lack of a child-appropriate formulation has been a glaring omission. This formulation development project goes some way to rectify a major inadequacy in current treatment options.

The present study investigated the PK characteristics of the novel oral liquid formulation of 13-CRA and capsule-extracted 13-CRA. The model estimated a 65% higher mean bioavailability for the oral liquid and implies a marked improvement in dose delivery. Previous studies have also shown that children able to chew and/or swallow intact 13-CRA capsules are significantly more likely to achieve target concentrations than those who have the drug extracted from capsules prior to administration. Veal et al. (2013) observed a significantly higher mean (SD) Cmax value of 1200 (660) ng/mL in patients who swallowed capsules as compared to 780 (545) ng/mL in patients who required the drug to be extracted prior to administration (*p* = 0.0012) [6]. They also noted that a target Cmax of 600 ng/mL was achieved by 93% (25/27) versus 55% (42/76) of patients in the two groups, respectively. In the present study, 80% (16/20) of patients exceeded a Cmax of 600 ng/mL with the oral liquid, compared to 45% (9/20) of patients with the capsule-extract method. Similarly, Gota et al. (2016) also showed that children who swallowed intact 13-CRA capsules (n = 18) achieved higher AUC_0–6h_ values compared to those who could not (n = 16); mean AUC 6470 vs. 2810 ng/mL·h (*p* < 0.05) [14]. Cho et al. (2017) assessed the PK of 13-CRA and 4-oxo-13-CRA (thought to be equally pharmacologically active) in Children’s Oncology Group (COG) phase III studies that treated HRNBL patients with 13-CRA and immunotherapy [15]. Of 617 patients, 370 (60%) achieved median combined plasma concentrations of 13-CRA + 4-oxo-13-CRA >1500 ng/mL. Plasma levels were higher in patients taking intact capsules relative to open capsule takers (13-CRA: 522 vs. 310, 4-oxo-13-CRA: 2160 vs. 990 ng/mL, respectively, *p* < 0.001). 

One possible explanation for why capsule-extracted 13-CRA results in under-dosing (lower systemic exposures) may be that the drug is not uniformly distributed in the capsule vehicle, with potentially some drug adsorbed onto the inner surface of the gelatin capsule. Alternatively, it may be related to the technical dexterity and experience of the person extracting the drug from the capsule, commonly the parent of the child being treated. Either way, the current situation is clearly unacceptable when multiple studies from different countries have highlighted the reduced 13-CRA drug exposures being achieved due to the limitations of the available drug formulation. 

The increased relative bioavailability of the newly developed liquid has implications for dosing in practice. The standard protocol dose of 13-CRA for patients swallowing intact capsules is 160 mg/m^2^. In a HRNBL setting in the UK and Europe this dose is increased to 200 mg/m^2^ for patients unable to swallow intact capsules, based on earlier studies that revealed lower exposures for capsule-extracted 13-CRA [6,7]. This increased dose was deemed necessary to replace inherent loss of drug when extracting from a capsule in order to achieve comparable exposures in all patients. The improved bioavailability of the new liquid formulation suggests that the delivered dose is more accurate and therefore a daily dose of 160 mg/m^2^, commensurate with the intact capsule dose, is appropriate. Nevertheless, the study confirms previous observations of significant inter-subject variability in exposures; Cmax and AUC concentrations ranged from 200–1200 and 250–2500 ng/mL, and 250–1000 and 400–1500 ng/mL·h, with the extracted capsule and liquid formulations, respectively. The observation that variability in systemic drug delivery was not reduced with the liquid formulation suggests that it is the physicochemical characteristic of 13-CRA that is most influential rather than formulation per se. Since dosing with 13-CRA is not adjusted according to blood levels, inherent variability in dose delivery runs the risk of under- and over-dosing with either formulation. However, there are limited clinical data investigating the relationship between plasma 13-CRA data and clinical outcomes, or indeed the optimum therapeutic plasma concentration range.

The current protocol dose of 160 mg/m^2^/day was determined from PK studies that demonstrated plasma concentrations in the region of 1500 ng/mL, the concentration at which in vitro models show a halt in the growth of neuroblastoma cell lines [16]. The two aforementioned PK studies also suggest, based on analysis of uncontrolled data, that systemic exposure is correlated with disease relapse. Gota et al. (2017) noted that children who were event-free at one year tended to have higher 13-CRA AUC compared to those who progressed or died, although this was a post-hoc analysis and was not statistically significant [14]. Cho et al. (2017) assessed the relationship of PK to overall survival (OS) in 13 CRA-treated patients [15]. In patients ≥18 months old at diagnosis (*n* = 445/524) the 5-year OS was significantly higher for patients with upper quartile 13-CRA levels (750 ng/mL, 73%) relative to lower quartile (180 ng/mL, 60%, *p* = 0.039), although event-free survival was not significantly different (*p* = 0.44). Higher active metabolite concentrations (4-oxo-13-CRA > 1500 ng/mL, 76%) were also associated with significantly higher OS relative to lower levels (<300 ng/mL, 66%, *p* = 0.032). 

Overall, the incidence of drug-related adverse events was as anticipated in this population of patients (mainly cheilitis, skin rash, dry skin, conjunctivitis), no greater than Grade 3 and all described as mild by Day 29. Two SAE’s were temporally related to the administration of oral 13-CRA liquid. In the case of acute renal failure (raised serum creatinine), the investigator confirmed that concomitant medication gabapentin or acyclovir or both could possibly be the likely cause of this SAE since they can both cause acute renal failure. The patient responded to intravenous fluids and the severity was graded as 1. In the case of hematemesis, the investigator assessed the event of upper gastrointestinal bleed with underlying varices as possibly related to administration of oral liquid 13-CRA. Although the investigator considered portal hypertension as the most likely cause, streaks of bleeding with vomit could also be secondary to dry/friable mucus and membranes and low platelets (<100). The bleeding resolved and again the severity was graded as 1.

Previous studies have observed that Grade 3 or 4 toxicity is related to peak plasma concentrations > 3000 ng/mL, higher than the maximum peak concentration of 2440 ng/mL observed in this study with the oral liquid formulation. There was also no evidence that higher mean systemic exposure with the novel oral liquid resulted in increased rates of all adverse events, although the sample size is small and therefore no definitive conclusions can be drawn. The latter point is particularly relevant bearing in mind the significant overlap in observed systemic exposures. 

Neuroblastoma is the most common cancer in babies and 90% of cases occur in children less than 5 years of age [1]. Therefore, the availability of a child-friendly formulation is of paramount importance. However, the development of an oral liquid formulation of 13-CRA is not trivial: 13-CRA readily oxidizes and isomerizes in the presence of light or excessive heat, posing significant difficulties in handling and processing of raw material, manufacturing and analytical testing. It is also virtually insoluble in water. To overcome these challenges, bespoke processes were developed for receipt, pack down and storage of bulk material, manufacturing (in an isolator) and analytical testing under blocked UV light and bottle filling under nitrogen purging. The final formulation developed is a stable, multi-dose, ready-to-use, child-friendly preparation of 13-CRA. It has a shelf-life of 18 months and an in-use shelf-life of 4 weeks when stored below 25 °C. Due to the lack of a commercial opportunity, the formulation will be made available to pediatric oncology centers as an unlicensed “special”.

The palatability and acceptability assessment confirmed that the oral liquid had good palatability rating, but also made daily dose administration much easier and convenient compared to the elaborate multi-step process necessary with capsules. The burden of accurately dosing and administering daily 13-CRA when extracted from capsules can only really be described by those intimately involved in this practice. Many healthcare professionals would struggle to perform this task even under ideal conditions. A ready-to-use oral liquid is not only much more convenient, but also minimizes the teratogenic risk for women of childbearing potential, with the current method requiring manipulation of capsules.

Optimizing dosing regimens of anticancer drugs for children presents a major challenge in the clinical oncology setting. This is particularly evident in infants and very young children due to the considerable developmental physiological changes occurring in this age group. Anticancer drugs are generally associated with large between-patient variability in PK and pharmacodynamics, in addition to the considerable effect of pharmacogenetics. It is generally accepted that a significant contributor to the failure of treatment in cancer chemotherapy, despite selection of the correct drug(s), is the failure to select the correct dose [16]. Chemotherapeutic drugs are typically adjusted for body size, but it is in general a one-size-fits–all approach. Personalized dosing through “therapeutic drug monitoring” feedback or Bayesian forecasting algorithms has been shown to improve clinical outcomes with chemotherapeutic drugs, but with oral therapy this is only achievable in children when there is a formulation that permits flexible dosing [17,18,19,20]. Importantly, the availability of a ready-to-use oral liquid will also allow doses to be personalized to young children using an oral syringe. As yet, personalized dosing of 13-CRA in the treatment of neuroblastoma does not have any supporting clinical outcomes data.

## 5. Conclusions

A novel oral liquid formulation of 13-CRA has been developed and evaluated in an open label PK, safety and palatability study. It can be concluded that the oral liquid compares favorably to 13-CRA extracted from the licensed capsule formulation. The population PK model shows that the administration of the liquid formulation significantly improves bioavailability, although there remains significant inter-patient variability and overlap of systemic exposures. Higher bioavailability increases the likelihood of achieving target systemic exposures, which in previous studies has proven to be challenging when 13-CRA is extracted from a capsule. However, the clinical significance of improved bioavailability is unknown and needs to be investigated in a larger clinical trial.

## Figures and Tables

**Table 1:** Demographics of the study population.

Characteristics (*n* = 20)	Statistic	Value
Age (years)	Median (range)	4.3 (1–11.6)
Sex Male/Female		15 (75%)/5 (25%)
Race		
White	n (%)	15 (25%)
Asian or Asian British	n (%)	4 (20%)
Other	n (%)	1 (5%)
Height (m)	Median (range)	100 (74–152)
Weight (kg)	Median (range)	14.2 (9.3–38.9)
BMI (kg/m^2^)	Median (range)	15.8 (13.4–18.2)
Sequence of formulation administration (A or B) ^1^		A = 11 B = 9
Dose (mg/m^2^)	Mean (SD)	192.1 (23.6)

^1^ A = subjects received liquid formulation first, B = subjects received capsule-extracted formulation first.

**Table 2:** Summary of drug-related adverse events.

Adverse Effect *	13-CRA Oral Liquid (*n* = 20)	13-CRA Extracted Capsule (*n* = 20)
	Number (%) of patients reporting	Number (%) of patients reporting
Dry eye	3 (15%)	2 (10%)
Chapped/dry lips	8 (40%)	6 (30%)
Dermatitis (exfoliative)	1 (5%)	0
Dry skin	8 (40%)	9 (45%)
Rash (maculo-papular)	1 (5%)	0
Skin exfoliation	7 (35%)	4 (20%)
Diarrhea	1 (5%)	1 (5%)
Hematemesis **	1 (5%)	0
Vomiting	2 (10%)	1 (5%)
Headache	0	1 (5%)
Acute kidney injury **	1 (5%)	0
Epistaxis	1 (5%)	0

* Severity of all AE’s was graded ≤ 3; ** Reported as SAE.

**Table 3:** Palatability and Acceptability.

Parameter		13-CRA Oral Liquid	13-CRA Extracted Capsule
Taste (mm) *		81.7 (9.6) *N* = 7	77.3 (17.7) *N* = 6
Smell (mm) *		80.8 (19.3) *N* = 6	73.7 (15.2) *N* = 6
Easy to take **	Not at all easy	0	3 (18.8%)
	Easy sometimes	0	4 (25.0%)
	Easy most of the time	2 (14.3%)	1 (6.3%)
	Easy all of the time	12 (85.7%)	8 (50.0%)
Ease of preparation and administering **	Extremely difficult	0	6 (40.0%)
	Very difficult	0	1 (6.7%)
	Difficult	0	2 (13.3%)
	Not easy	1 (7.7%)	2 (13.3%)
	Quite easy	3 (23.1%)	2 (13.3%)
	Easy	2 (15.4%)	1 (6.7%)
	Very easy	7 (53.8%)	1 (6.7%)

* Mean (SD) visual (hedonic) analogue scale ranging from 0 (‘Bad’) to 100 (Good) mm. ** Number of subjects (% of total) responding to question.

## Data Availability

The datasets generated and/or analyzed during the study are not publicly available due to proprietary restrictions but are available from the corresponding author on reasonable request.

## Supplementary Materials

The following are available online https://www.mdpi.com/article/10.3390/cancers13081868/s1, File S1: Pharmacokinetic Modelling Methodology, Figure S1: structure of the parent–metabolite model, Figure S2: visual predictive checks, Figure S3: NPDE goodness of fit plots from the final model for the liquid formulation of 13-CRA, Figure S4: NPDE goodness of fit plots from the final model for the capsule-extracted 13-CRA, Table S1: model parameters.
