# Supplementary material for: Pharmacokinetics and Safety of a Novel Oral Liquid Formulation of 13-cis Retinoic Acid in Children with Neuroblastoma: A Randomized Crossover Clinical Trial
Source: Cancers (Basel). 2021 Apr 14;13(8):1868. doi: 10.3390/cancers13081868 (PMC8070696; doi:10.3390/cancers13081868)
Supplement: Supplementary file 1 [file cancers-13-01868-s001.pdf]

Supplemental File:

Pharmacokinetic Modelling Methodology and Results

Base model

It was assumed that all 13-CRA that is absorbed is metabolized to 4-oxo-13-CRA before being excreted.

The base model was parameterized using volumes and clearances. The difference of individual parameters P_i from the population mean ϑ (inter-individual variability, IIV) was parameterized as η and described using one of the following equations:

$P_i = \theta + \eta$ Constant (additive) variance model

$P_i = \theta \cdot \exp(\eta)$ Exponential (lognormal) variance model

The residual variability, which is the result of assay error, data error and model misspecification was modelled using one of the following equations:

$C_{ij} = C_{pred,ij} + \varepsilon_{ij}$ Additive error model

$C_{ij} = C_{pred,ij} \cdot (1 + \varepsilon_{ij})$ Proportional error model

Where C_{ij} is the jth observation of the ith individual, $C_{pred,i,j}$ is the model-predicted value and ε_{ij} is the residual error for the current observation. All variability parameters were characterized by assuming normal distributions with a mean of 0 and an estimated variance of ω^2 for IIV and σ^2 for residual error.

The strategy was to first develop a popPK model for 13-CRA only, the incorporation of absorption, transit compartments and further parameters for different formulations were explored. Once a satisfactory model was identified, a second model for 4-oxo-13-CRA was developed. The two models were then combined and inferred onto the 13-CRA and 4-OIT data as a whole. Covariance between parameters for the 13-CRA part and for the 4-OIT part of the model were investigated and further refinements were made until a combined base model was identified that satisfied all model selection criteria.

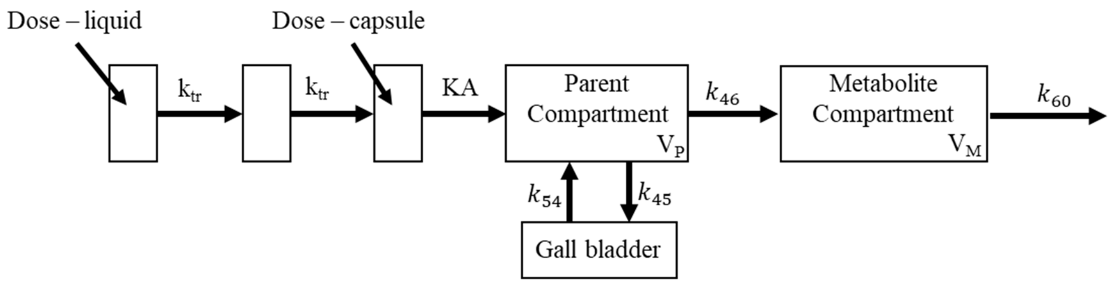

FigureS1: Structure of the 13-CRA parent-metabolite model

Covariate model

Age, sex, various scalars of body size (weight, height, BSA, BMI) and formulation sequence were investigated on all structural PK parameters with IIV in the popPK base model.

The covariate effects were implemented in the base model as follows:

- Allometric scaling was done for bodyweight:

$$P_i = \theta_{TV} \cdot \left(\frac{BW_i}{BW_{med}}\right)^{\theta_{cov}} \cdot \exp(\eta)$$

- Continuous covariates

$$P_i = \theta_{TV} \cdot \exp\big(\theta_{cov} \cdot (COV_i - COV_{med})\big) \cdot \exp(\eta)$$

- Categorical covariates

$$P_i = \theta_{TV} \cdot (1 + N1 \cdot \theta_{cov1} + N2 \cdot \theta_{cov2} + \cdots) \cdot \exp(\eta)$$

  Where P_i denoted the individual structural parameter value, θ_{TV} the estimated typical parameter value, BW_i the individual's bodyweight, BW_{med} the population median bodyweight. COV_i and COV_{med} are the individual and population median covariate values, respectively. θ_{cov} the estimated parameter for the covariate effect. N1=N2=0 for the most prevalent covariate category, N1=1 and N2=0 for the next most prevalent category, and N1=0 and N2=1 for the next category etc. The number of terms in the equation depends on the number of covariate categories.

The covariate analysis was performed with a full stepwise forward inclusion/backward elimination procedure. During forward inclusion, a reduction in the objective function value (OFV) corresponding to $p < 0.01$ (ΔOFV<-6.67 for one estimated parameter, with adjustment for greater than one parameter according to Chi-squared distribution) was required for the declaration of a significant covariate effect. In backward elimination, the requirement was increased to $p < 0.001$ (ΔOFV>+10.828 for one estimated parameter). The covariate model after the forward inclusion and backward elimination was called the selected popPK model.

The influence of the maturation of renal and enzymatic function on the clearance on 13-CRA and 4-OIT was not investigated due to only 10% of patients being ≤ 2 years old.

Terminal elimination half-life was not calculated due to lack of data during the terminal phase making predictions unreliable.

Model qualification

The main tool used for model qualification was the prediction-corrected VPC. These were generated from 1000 repeat simulations of the entire dataset with all random variables (inter-individual and residual) being sampled. The prediction-correction afforded an effective diagnostic display across a wide variety of doses and covariates. Within each time bin, the 5th, 50th, and 95th percentiles of the prediction-corrected observed and simulated data are calculated. From the 1000 replicates, a non-parametric 90% confidence interval for each of the three percentiles of the simulated data is obtained.

Prediction of individual exposure variables

Individual exposure variables at steady state for 13-CRA ($Tmax_{ss}$, $Cmax_{ss}$, $AUC_{(0\text{-}12)ss}$) and 4-oxo-13-CRA ($Cmax_{ss}$, $AUC_{(0\text{-}12)ss}$) were calculated after seven simulated doses using numerical integration of the popPK model.

Visual Predictive Check

The VPC of the final model was stratified according to the formulations for 13-CRA and according to day for 4-oxo-13-CRA (Figure S2). The VPC plots show that the model is able to explain both the central tendency and variability in the PK observations for 13-CRA, for both the new liquid and reference capsule-extracted 13-CRA. The VPC also demonstrate that the model is able to adequately explain the 4-oxo-13-CRA data on day 1, however there is a slight overestimation of the 95th percentile of 4-oxo-13-CRA data. The extent of the overestimation is small and is not considered to invalidate the model. Nevertheless, caution should be used when simulating steady-state profiles for 4-oxo-13-CRA.

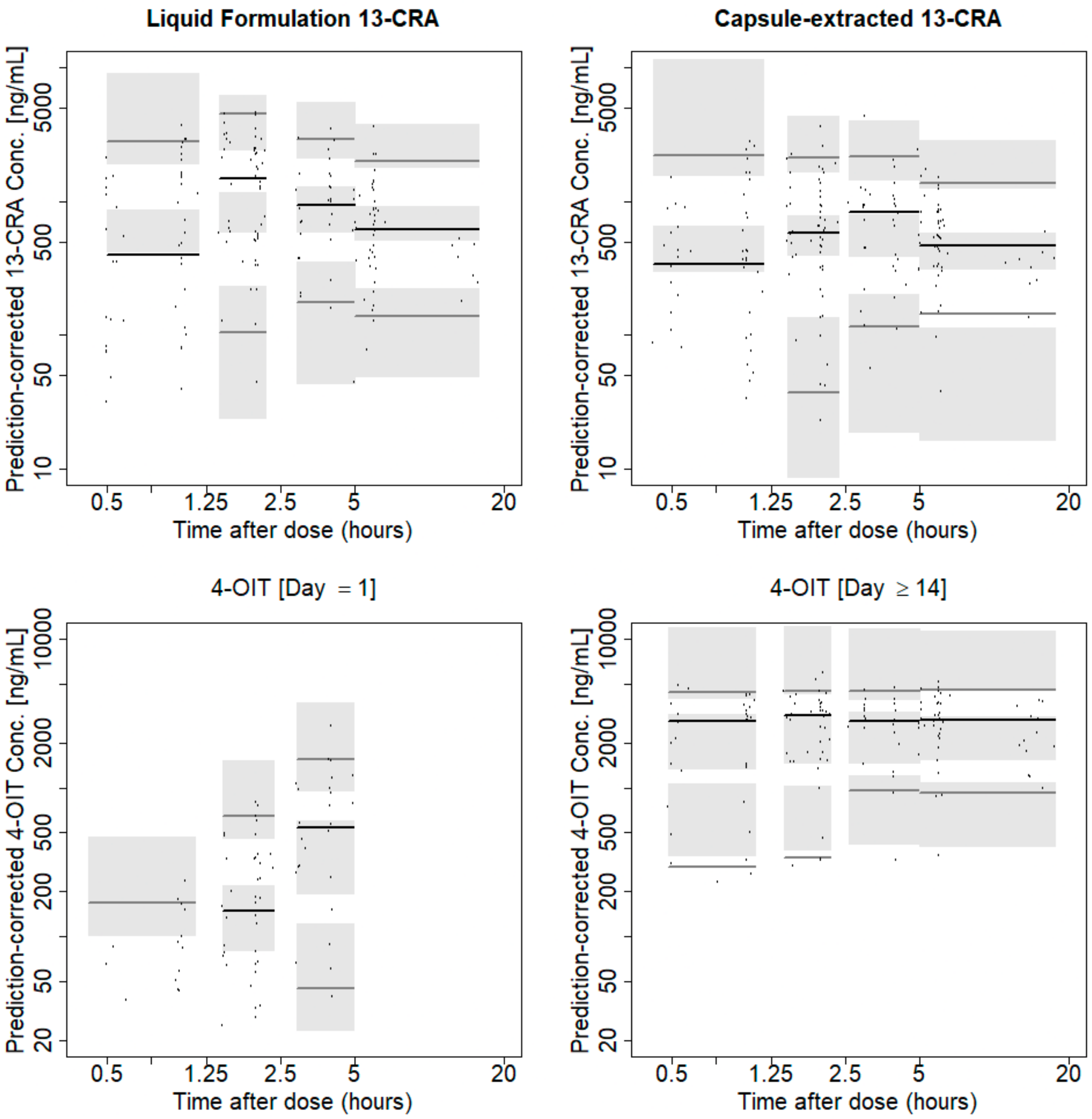

Figure S2 : VPC plots for liquid and capsule: 13-CRA (upper panel) and 4-oxo-13-CRA (lower panel). The VPC plot shows grey and black horizontal lines depicting the 5th, 50th and 95th percentiles of the prediction-corrected observed data in each bin, along with shaded regions depicting the limits of the 90% confidence intervals around the 5th, 50th and 95th percentiles of the prediction-corrected simulated data.

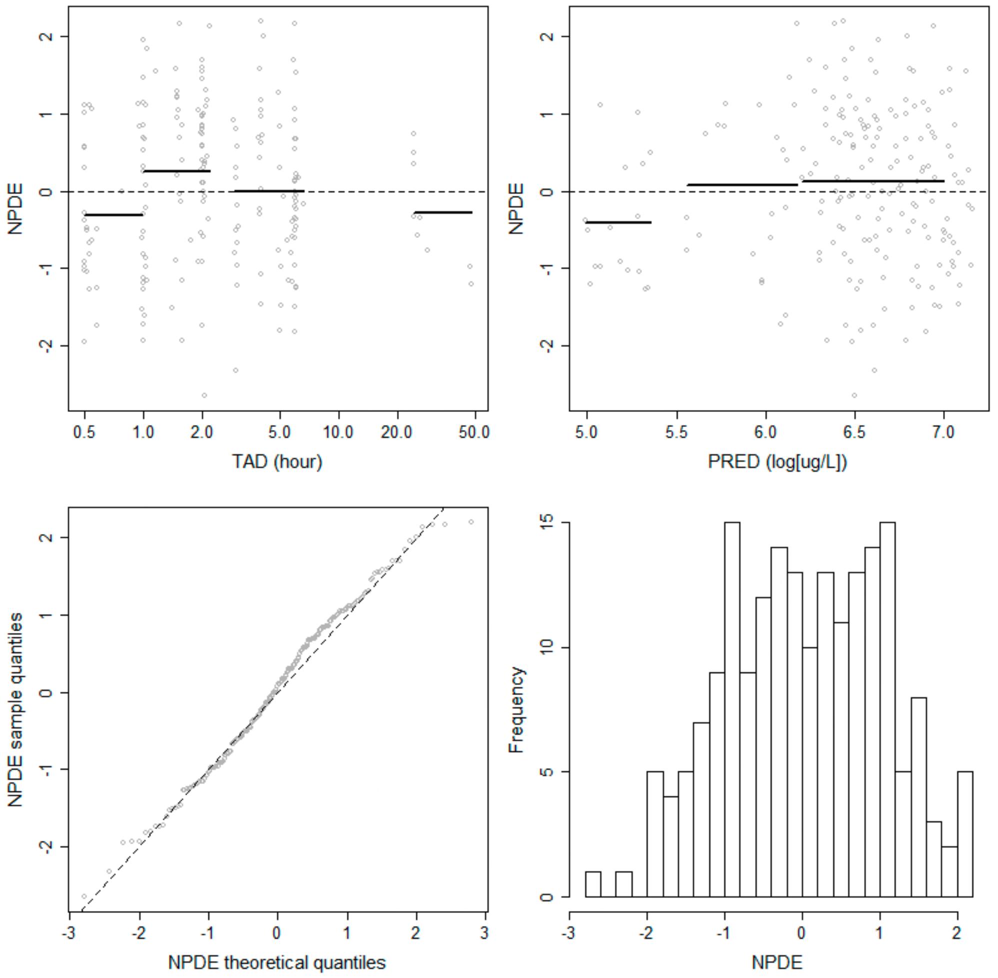

Figure S3: NPDE goodness of fit plots from the final model for the liquid formulation of 13-CRA. Dashed lines show lines of identity, bold horizontal lines show mean of NPDE in each bin of independent variable.

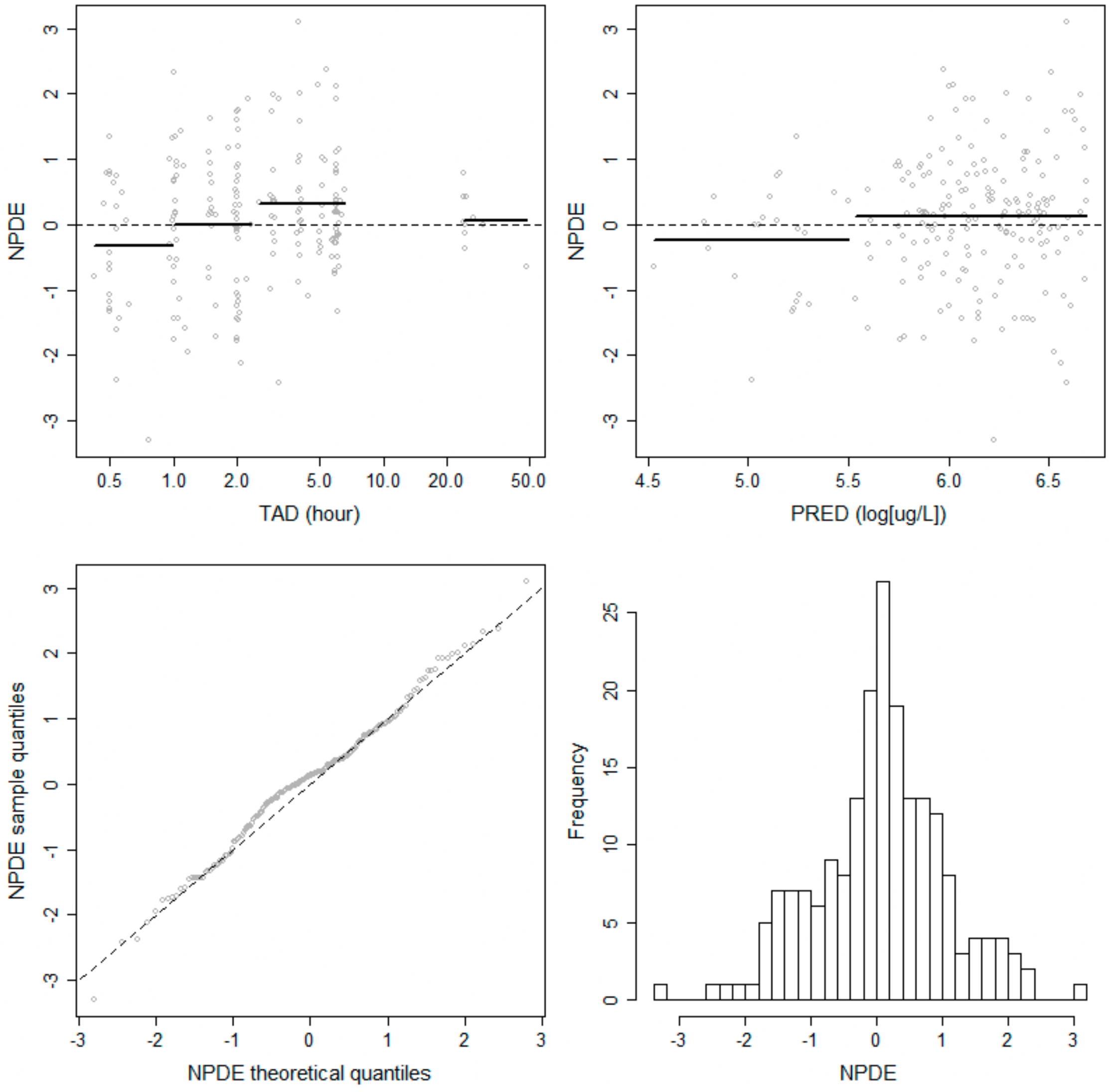

Figure S4: NPDE goodness of fit plots from the final model for the capsule-extracted 13-CRA. Dashed lines show lines of identity, bold horizontal lines show mean of NPDE in each bin of independent variable.

Table S1: Model Parameters

Parameter	Unit	Estimate	RSE [%]	LLCI	ULCI	Description
Fixed effects (THETA)						
k_{tr}	h^{-1}	9.23	27.002	4.345	14.115	Transit compartment rate constant for typical patient administered liquid formulation
KA	h^{-1}	0.249	63.606	-0.061	0.559	Absorption rate constant for typical patient
[1]CL_P^{pop}	L/h	9.74	30.294	3.957	15.523	Clearance of 13-CRA for a typical patient
[1]V_P^{pop}	L	41.15	--	--	--	Volume of distribution of 13-CRA for a typical patient
[1]CL_M^{pop}	L/h	2.66	56.809	-0.302	5.622	Clearance of 4-OIT for a typical patient
[1]V_M^{pop}	L	30.3	29.658	12.686	47.914	Volume of distribution of 4-OIT for a typical patient
F1		1.65	4.204	1.514	1.786	Relative bioavailability of liquid formulation with respect to the extracted capsule
k_{45}	h^{-1}	0.1047	--	--	--	Rate constant governing simplified enterohepatic recycling model
k_{54}	h^{-1}	0.05652	--	--	--	Rate constant governing simplified enterohepatic recycling model
Random effects: Inter-individual variability (OMEGA) diagonal elements						
V_P (ω^2)	-	1.6	166.231	-3.613	6.813	Inter-individual variability on V_P
V_P (CV)[2]	%	198.82				
V_P (Sh)[3]	%	4.87				
CL_P (ω^2)	-	0.141	110.279	-0.164	0.446	Inter-individual variability on CL_P
CL_P (CV)[2]	%	38.91				
CL_P (Sh)[3]	%	5.25				
CL_M (ω^2)	-	0.392	106.415	-0.426	1.21	Inter-individual variability on CL_M
CL_M (CV)[2]	%	69.28				
CL_M (Sh)[3]	%	5.16				
Random effects: Inter-individual variability (OMEGA) off-diagonal elements						
V_P and CL_P (ω^2)	-	0.346	175.242	-0.842	1.534	
V_P and CL_M (ω^2)	-	0.517	189.908	-1.407	2.441	
CL_P and CL_M (ω^2)	-	0.23	103.901	-0.238	0.698	
Residual error (SIGMA)						
Err_{parent} (σ^2)	-	0.39	17.521	0.256	0.524	Variance of exponential residual error for 13-CRA.
Err_{parent} (CV)[4]	%	69.06		54.01	82.99	
Err_{parent} (σ^2)	-	46656	19.44	17875.4226	88983.4866	Variance of additive[6] residual error for 13-CRA.
Err_{parent} (CV)[5]	%	21.6		13.37	29.83	
$Err_{metabolite}$ at Day=1 (σ^2)	-	0.793	14.386	0.569	1.017	Variance of exponential residual error for 4-OIT on day 1
$Err_{metabolite}$ at Day=1 (CV)[4]	%	110		87.55	132.85	
$Err_{metabolite}$ at Day=14 (σ^2)	-	0.139	21.206	0.081	0.197	Variance of exponential residual error for 4-OIT on day ≥ 14

$Err_{metabolite}$ at Day=14 (CV)[4]	%	38.62		29.05	46.66	

LLCI = lower limit of 95% confidence interval (estimate – 1.96·SE)
ULCI = upper limit of 95% confidence interval (estimate + 1.96·SE)
RSE = relative standard error (100·SE/estimate)
[1] Apparent PK parameter, equivalent to CL_{pop}/F and V/F, where F is the absolute bioavailability of the capsule formulation.
[2] The coefficient of variation (CV) is calculated as 100·SQRT(EXP(OMEGA^2)-1)
[3] Shrinkage (Sh) calculated as 100·(1-standard deviation of individual eta estimates/ω)
[4] The coefficient of variation (CV) is calculated as 100·SQRT(EXP(OMEGA^2)-1)
[5]The coefficient of variation (CV) is calculated as 100·SQRT(SIGMA^2)/1000, which is the CV for an observation of 1000 ng/mL.
[6]This parameterization of the residual error model approximates an additive structure on the untransformed scale.

k_{tr}, transit compartment rate constant, KA, absorption rate constant; V_p and V_M volume of distribution for the central compartments of the parent (13-CRA) and the metabolite (4-OIT), respectively; k_{45} and k_{54}, are the rate constants between central and peripheral compartments for the parent; k_{46} and k_{60}, are the elimination rate constants for the parent and the metabolite; CL_P and CL_M, respectively are the clearance for the parent and the metabolite while CL_P^{pop}, CL_M^{pop}, V_P^{pop} and V_M^{pop}, respectively are the estimated clearance and volume of distribution for a typical patient.
